# Preliminary examination of a mentor-based program for eating disorders

**DOI:** 10.1186/s40337-014-0024-0

**Published:** 2014-08-22

**Authors:** Marisol Perez, Ashley Kroon Van Diest, Shannon Cutts

**Affiliations:** Department of Psychology, Texas A&M University, 4235 TAMU, College Station, TX 77843-4235 USA; MentorCONNECT, www.mentorconnect-ed.org, Houston, Texas USA

**Keywords:** Mentor program, Eating disorders, Quality of life, Treatment compliance

## Abstract

**Background:**

There is a current and pressing need for recovery resources for individuals suffering from eating disorders. Mentoring programs have been useful with other psychiatric disorders such as addictions, and may be useful for individuals recovering from an eating disorder. The present study sought to examine a mentoring program for individuals working towards recovery from an eating disorder.

**Methods:**

The study included mentors (i.e., individuals who have recovered from an eating disorder for an extended period of time), and mentees (i.e., individuals who were in the process of recovering from an eating disorder and wanting additional support aside from their treatment team). Participants included 141 participants, consisting of 34 mentors, 58 mentees who matched with a mentor, and 49 mentees searching for a mentor. Participants completed questions assessing eating disorder symptoms, quality of life, motivation towards recovery, and treatment compliance.

**Results:**

Matched mentees reported higher levels of quality of life on 7 out of 12 domains, and missed fewer appointments with treatment providers when compared to unmatched mentees. There were no differences between matched and unmatched mentees on motivation, energy or confidence towards recovery.

**Conclusions:**

Findings suggest a mentor model is beneficial for individuals engaged in the process of recovering from an eating disorder in the areas of quality of life and treatment compliance. Specifically, mentees in a mentoring relationship reported better family and close relationships, future outlook, and psychological, emotional, and physical well-being than unmatched mentees. Mentors reported being positively impacted by the mentoring relationship by strengthening the skills they learned while in recovery, and reminding them of how far they had come in their own recovery. The findings in this study suggest that mentor programs warrant further investigation as ancillary support services for individuals recovering from an eating disorder.

## Background

A recent article by Kazdin & Blasé [1], argues that psychotherapy and clinical practice need a reboot, with the current model of individual psychotherapy unable to significantly reduce the burden of mental illness. The authors suggest that in order to better reduce the burden of mental illness there needs to be a greater array of services available that maximize technology, such as the internet, and expand opportunities for non-professionals to assist in prevention and support services [1]. Kazdin & Blasé’s [1] article accurately reflects the field of eating disorders, where there is a current and pressing need for recovery resources for individuals suffering from eating disorders [2,3]. In a recent study of eating disorder services, both patients and carers mentioned support services as one of the top five essential features of high quality eating disorder services [2], yet report a lack of services available [3]. In addition, 294 individuals recovering from an eating disorder reported support during all stages of the recovery process as important for recovery, the need for additional support outside of psychological treatment, and financial costs being the biggest barrier to services [2,3].

Affordable ancillary support services for individuals wanting to recover from an eating disorder have the potential to ease some of the financial burden and the pressing need for support during the recovery process [2,3]. Interviews with individuals recovered from an eating disorder indicate that non-professional contacts such as support groups are important components of the recovery process [4]. Ancillary support services can range from local community support groups for carers and sufferers of eating disorders [5–9], to support groups for co-occurring issues such as Alcoholics Anonymous [10] or Overeaters Anonymous [11], nutritional assistance [12], and online resources [13–15]. However the research on ancillary support services for individual suffering from eating disorders is sparse, with limited information on how these services can assist in the recovery process. For example, there are several studies that have assessed the benefits of support groups, but most have been conducted via interviews from the patient’s perspective [5–9] with no objective assessments used to ascertain the success or benefits of the groups. In addition, the literature on online eating disorder support groups has focused on thematic content analysis of the discussions [13,14]. A paucity of studies have examined community support groups for eating disorders with co-occurring issues such as Overeaters Anonymous and found these groups to be beneficial and significantly reduce symptoms [11,16]. Given the limited research on ancillary support services and the pressing need for low-cost services, the current study seeks to improve upon the literature by empirically examining the benefits of an additional ancillary support service, a mentor-based program developed for individuals recovering from an eating disorder that is free. The mentor-based program was designed to provide additional support and accountability in-between sessions for individuals in treatment, or keep individuals active in the recovery process if they were not in treatment [17].

Using a mentor model to facilitate recovery in mental health is not a novel concept. The most recognized mentor-type model is the Twelve Step model used in the fellowship of Alcoholics Anonymous [10]. The mentor-mentee model revolves around the willing presence of a volunteer who has achieved the “goal,” working in partnership with a person who is aiming to achieve the goal. Straussner and Byrne [18] undertook a review of the benefits of Alcoholics Anounymous and found several studies demonstrating the efficacy of the mentor-mentee program in reducing the mentee’s drinking. In addition there is research from Alcoholics Anonymous programs to demonstrate that the mentorship relationship is also beneficial for the mentor with the mentoring relationship helping the mentor stay recovered [19–21]. These findings collectively suggest that the mentor-mentee model of Alcoholics Anonymous benefits both the mentor and mentee in their recovery from alcohol.

The mentor model is also used in many health/behavioral areas including school programs, business, mental health and infant health [22–24]. A review of the organizational and vocational literature for the past 25 years found a positive impact of mentorship for the mentee, mentor, and the organization [22]. In addition, a recent review of school programs where mentors were adults who met with youth mentees, found that mentor programs increase a youth’s connectedness with school, family, and community, and reduces dropout rates and problematic behavior in the classroom [23]. Finally, a recent mentor model was implemented with success to facilitate the detection and introduction of services for infant mental health problems by direct medical providers, where the mentors were mental health professionals who specialized in infant mental health [24].

A review of the empirical literature on eating disorders revealed that there are no studies discussing a mentor-mentee program for individuals recovering from an eating disorder. Given the success of the mentor model for other vocational, education, and mental health areas outside of the eating disorders field, a mentor program for eating disorders may have several potential benefits to patients and the field. Some individuals in outpatient treatment would benefit from regular, sustained contact with additional recovery-based support and accountability resources beyond what professional treatment sessions can offer to them [13,14,17]. Reviews of discussions on electronic support groups has found that individuals suffering from eating disorders seek emotional support, and information and feedback, with members with longer periods of recovery serving as role models [13,14]. A mentor program can also assist patients in coping with triggers or negative life events, and decreases in motivation that occur in between therapy sessions [17]. For those contemplating entering treatment or those individuals who do not have access to treatment, a mentor program can offer some form of recovery-based support and serve as a motivator for finding and maintaining treatment [17]. For the field of eating disorders, a mentor program can provide additional no-cost support services that can reduce the financial burden reported by patients that serves as a barrier to needed care [2,3]. Thus, a mentor program for individuals suffering from eating disorders can have numerous potential benefits to the individual sufferer and the field.

The field of eating disorders has debated the role of the recovered individual in the recovery process of others suffering from an eating disorder [25]. Concerns with having recovered individuals involved in the recovery process of others revolve around countertransference issues, taking over the recovery process for the patient, and risk of relapse [25,26]. On the other hand, reports from treatment providers with a history of an eating disorder [26], recovered individuals who have been symptom free for at least 1 year [4], and research from online support groups [13] and Overeaters Anonymous [11] suggest that there are benefits to having recovered individuals involved in the treatment of others. In addition, mentor models are based on the helper therapy principle, which states that there are intrinsic rewards and benefits to helpers when they assist others, including maintaining treatment outcomes [27]. The current study will contribute to this debate by examining if there are benefits to engaging in a mentor relationship for individuals who are in the process of recovery. In addition, we examine the perspectives of the recovered individual who are serving as mentors.

The current study sought to improve upon the ancillary support services literature in 3 ways: 1.) examine the benefits of a mentor model for individuals recovering from an eating disorder for which there is no previous research; 2.) use common, empirically validated, self-report questionnaires deviating from the existing literature that uses interviews and online discussions with thematic content analysis; and 3.) provide the perspective of the mentor and the needs of the sample which is also limited in the existing literature. It was hypothesized that mentees who were working with a mentor, would have more motivation to recover from an eating disorder, spend more time and energy devoted to recovery work, have a higher reported quality of life particularly in the arena of interpersonal relationships, and perhaps exhibit greater treatment compliance than mentees who were searching for mentors but had not yet found one. The current study also sought to quantify specific topics that mentors and mentees regularly discussed, to describe the most common additional support needs that mentees reported, and to ascertain the impact of mentoring on the volunteer mentors.

## Method

### Study design & procedures

The data collected from this study was part of a larger program evaluation designed to assess unmet need, service use and satisfaction with MentorCONNECT. This was a cross-sectional, retrospective study with the use of online self-report questionnaires. It is common for mentees that would like a mentor to search for several months for a mentor, with the main reason for not finding a mentor being availability (Shannon Cutts, personal communication, April 2011). During the time of the study, there were more mentees wanting mentor services than there were mentors available, creating a natural control group (i.e., people were not randomized into a control group or intervention group). These wait-listed individuals are labeled unmatched mentees in this study.

Prior to the study, all self-report questionnaires were reviewed by a board of 18 individuals including researchers, licensed professionals, and recovered individuals. Numerous board members were concerned with the self-reported weight item as potentially triggering for participants. Given the overwhelming and strong response to the weight item, it was dropped from the questionnaire. This study was reviewed and approved by the Texas A&M University’s Institutional Review Board, and all participants completed online consent forms and agreed to participate in the research study before completing the online survey. The online survey was accessible for a 2-month period, but participants could only participate once.

### Recruitment

**Figure 1:**
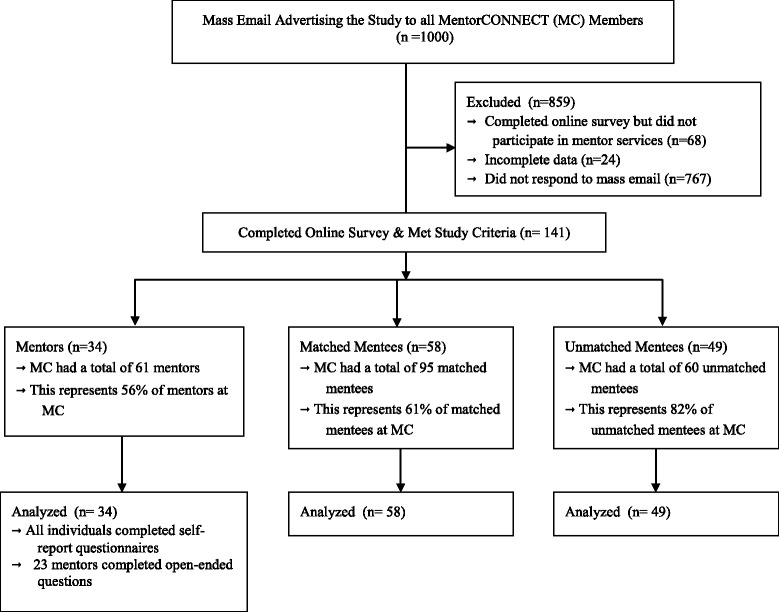
**CONSORT diagram.**

Participants were recruited through a mass email (approximate *N* = 1000) inviting them to participate in a 20-minute online survey over a 2-month period. All members and new members that joined MentorCONNECT during the 2 month study period were invited to participate. A total of 76.7% of the sample did not respond to the mass email with more details provided in Figure 1. It is unknown why only 23.3% of the targeted sample responded to the study. As an incentive to participate, survey participants who completed the survey were entered into a raffle to win numerous prizes related to recovery. Participation was completely voluntary.

### Participants

A total of 141 individuals participated in this study, consisting of 34 mentors, 58 matched mentees, and 49 unmatched mentees, see Figure 1 for more details. The racial composition of the entire sample was 81% White, 1% Black, 4% Hispanic, 2% Asian, and 5% other. A total of 9% of the sample was currently living outside of the United States. Participants ranged in age from 15 to 63, with a mean age of 31 (SD = 10.26). Mentors were on average 34 years old (SD = 12.88), and consisted of 4 mental health professionals with no history of an eating disorder, and 30 recovered individuals who reported being recovered for an average of 8 years (SD = 6.00). Almost all of the mentors (93.8%) were living in the United States at the time of this study and all were women. Mentors were asked what eating disorder they most closely identified with during the course of their illness and were allowed to endorse more than one disorder. Approximately 56% of mentors identified with anorexia nervosa, 38% bulimia nervosa, 15% binge eating disorder, and 38% eating disorder not otherwise specified. Mentees ranged in age from 19 to 59 years old with an average of 31 years old (SD = 8.94), and were predominantly women. Table 1 provides the descriptive information for matched and unmatched mentees.

**Table 1:** **Demographic information on matched and unmatched mentees**

	**Matched**	**Unmatched**	***t*** **or** ***Χ*** ^***2***^	***p***
Age (year born)	1982 (8.64)	1980 (9.34)	0.71	.48
International status	19%	20%	0.04	.85
Ethnicity (Caucasian)	90.9%	88.6%	3.38	.64
Self-reported diagnosis				
AN	62%	49%	1.85	.17
BN	29%	37%	0.67	.42
BED	10%	16%	0.84	.36
EDNOS	41%	35%	0.50	.48
EDDS total	1.77 (0.67)	1.50 (0.97)	1.65	.10
Length of time at MC			0.51	.92
Less than 3 months	33%	37%		
3 – 6 months	26%	29%		
7 – 12 months	22%	18%		
More than 1 year	19%	16%		

Note: International status refers to the percentage of the sample born outside the United States. Self-reported diagnosis – participants were asked to endorse the diagnosis they most closely identified with and could choose more than 1 disorder. For age born and EDDS total the Means (Standard Deviations) are reported. MC – *MentorCONNECT*; EDDS – Eating Disorder Diagnostic Scale. Matched mentee group *N* = 58; unmatched mentee group *N* = 49.

### Mentor program

The mentor-based program was created by *MentorCONNECT*, a nonprofit organization that is a global eating disorders-focused mentoring community. Individuals seeking additional support recovery services from eating disorder can become members of *MentorCONNECT.* All services are provided for free. Mentors were volunteers who were mental health professionals that specialize in eating disorders, and/or individuals who have personally achieved 12 consecutive months of sustained recovery, defined as “largely free from eating disordered thoughts and coping behaviors”. Mentor volunteers were pre-screened by a team of lay and licensed professionals to assess their recovery status before being approved to serve. Mentors receive a package of materials to assist with the mentoring process and are assigned an experienced mentor to assist them. For a review of this mentor model please see Cutts [17]. Each approved volunteer mentor writes a free-form paragraph about their personal recovery history, including why they want to serve as a mentor and what they have to offer, their availability, and preferred method of contact. This information is posted on a password-protected page that mentees can view to request a match with their preferred mentor. A mentor-level volunteer leader serves as the Mentoring Match Coordinator and assists mentees and mentors with finalizing a new match. Once a match is made, the new pair completes an Immediate Action Plan form that outlines their communication preferences and solidifies their commitment to regular communication and to the match itself. Pairs are asked to commit to communicating at least once per week. Mentors are asked to commit a minimum of one hour per week per mentee, up to a maximum number of three mentees per mentor.

The mentoring relationship within this program was modeled after past research from the organizational/vocational literature [28]. The first phase of the mentoring relationship is the initiation phase [28]. In this phase, the mentor and mentee get to know each other, decide if the mentor might be appropriate to meet the mentee’s needs, establish the boundaries of the relationship, and establish the mode and frequency of contact. During this phase it is also recommended that mentors review with mentees how both parties can respectfully end the relationship if they need or want to discontinue. During this phase mentors and mentees tend to report feeling both excitement and challenge as they start to work together (Shannon Cutts, personal communication, April 2011). The second phase of the mentoring relationship is the cultivation phase [28]. During this phase the mentor and mentee mutually benefit from and develop the relationship further depending on the needs and interest of both parties. The mentoring relationship can change dramatically during this time, particularly if mentees move into or out of inpatient treatment, day hospitalization, and outpatient treatment. The mentoring topics will also gradually shift over time from focusing on what the mentee is doing on a daily basis that is complying with treatment goals and recovery, towards relapse prevention, and increasing quality of life and life satisfaction issues. This phase is usually reported by both parties in a positive light [28]. Over time and through successful treatment and a continuing complementary evolution in the mentoring relationship, the mentee becomes more independent and is less dependent on the mentor, which leads into the third phase, the separation phase. During this phase, the mentor may report feeling not needed, or may feel that there is limited use made of the support they are continuing to offer. Although there may be feelings of sadness as the relationship transitions and often ends, this period is usually celebrated by both parties as a successful conclusion to a mutually productive relationship. It is important to note that the time frame of the mentoring relationship can vary by mentee. Some mentees only need support for a short period of time to help them get through a difficult time, while for others; the mentoring relationship may last several years. After the mentor and mentee decide to end their formal mentoring relationship, they enter into the final phase. In the redefinition phase, the pair can decide to end the relationship and cease communication or change the quality of the relationship to mirror more of a friendship [28].

### Measures

#### Group status

Participants were asked to identify how they used *MentorCONNECT* (i.e. membership level) with the options being, *mentor, matched mentee* (i.e., an individual working with a mentor)*,* and *unmatched mentee* (i.e., an individual searching or waiting for a mentor to come available). Participants’ response on this item determined their group status.

#### Eating Disorder Diagnostic Scale (EDDS)

The EDDS is a brief self-report measure of 22 items designed for diagnosing anorexia nervosa, bulimia nervosa, binge eating disorder, and eating disorder not otherwise specified based on the DSM-IV [29]. The EDDS items can also be standardized and summed for use as a continuous symptom composite scale. The EDDS has good psychometric properties [29,30]. For this study, the internal consistency using the standardized items was 0.95. As mentioned in the Procedure section, the self-reported weight item was removed from this scale. Thus, anorexia nervosa as defined by the DSM-IV could not be computed, instead sub-threshold anorexia nervosa was computed following the EDDS scoring protocol.

#### Eating Disorder Quality of Life Scale (EDQLS)

The EDQLS assesses functioning or quality of life among adolescents and adults suffering from eating disorders [31]. This measure contains 40 items that assess 12 domains of functioning considered to be important by patients suffering with eating disorders and consistent with the World Health Organization’s definition of quality of life: cognitive, education/vocation, family & close relationships, relationships with others, future outlook, appearance, leisure, psychological, emotional, values & beliefs, physical, and eating disorder (i.e., level of impairment in daily functioning directly due to an eating disorder). Items are rated on a 5-point scale ranging from *strongly disagree* to *strongly agree* with higher scores indicating higher levels of functioning. The EDQLS has demonstrated good psychometric properties [31]. Internal consistency of the EDQLS for this study was 0.97 for the total scale, 0.75 for the cognitive domain, 0.80 for education/vocation, 0.58 for family & close relationships, 0.77 for relationships with others, 0.76 for future outlook, 0.85 for appearance, 0.91 for leisure, 0.72 for psychological, 0.76 for emotional, 0.77 for values & beliefs, 0.57 for physical, and 0.89 for eating disorder.

#### Motivation, energy, & confidence towards recovery

Participants were asked “how motivated are you to recover?,” “how much energy do you spend on a daily basis towards recovery?,” and “how confident are you that you will recover?,” with all three questions using a 5-point scale where higher scores indicate more motivation, energy and confidence towards recovery.

#### Treatment compliance

Participants were asked, “In the past month, how many appointments with your treatment providers have you missed?” Prior to this question, treatment providers were defined as anyone on the individual’s treatment team. Participants entered the number of appointments missed in the past 30 days. Participants only completed this item once.

#### Length of mentoring relationship

Mentees were asked how long they had been in a mentoring relationship with a mentor from MentorCONNECT, with responses entered in number of months.

#### Frequency of communication

Both mentors and matched mentees were asked how frequently they communicated, with the options being *daily or almost daily, 2–3 times per week, once a week, twice a month or more*, and *once a month*.

#### The mentoring process

Mentors and matched mentees were asked what they discussed from a list of 27 topics: dealing with triggers from family and environment; managing or reducing eating disorder thought and behaviors; finding enough quality support services; lack of access to professional treatment; lack of willingness to seek treatment; lack of understanding or support from friends and family; support offered by family and friends isn’t what is needed; managing relationships with peers, family, friends, colleagues, society; asking for and accepting help from others; choose who to ask for help; managing relapses; body dissatisfaction and poor body image; setting and working towards recovery goals; dealing with weight stabilization; following a meal plan; grocery shopping/eating out/food-based socializing; learning to name, feel, and deal with emotions; finding time for recovery amidst other obligations; setting and honoring boundaries; choosing recovery; finding reasons to recover; using my voice and expressing needs, wants, desires, and boundaries; staying motivated and committed to doing the hard work of recovery; intimacy and relationship challenges, fears, and issues; finding and expressing preferences, skills, talents, and identity outside of “Ed”; building meaning and quality of life into the recovery process; other co-occurring recovery issues with addiction, trauma, abuse, self-harm, depression, anxiety, etc. Mentors were also given the opportunity to describe topics of communication, or provide more details about any of the topics listed above through open-ended questions.

#### Impact of program on mentors

Mentors were asked in an open-ended question what the impact of mentoring was on them. They were able to write up to 250 words to describe their experience.

#### Unmet need

Matched and unmatched mentees were also asked about current needs for which they would like assistance from a mentor.

## Results

Descriptive analyses were conducted comparing matched and unmatched mentees and there were no differences in age, international status, ethnicity, eating disorder diagnosis affiliation, and eating disorder symptoms total, see Table 1. There were no differences on income level across the groups with 69% of the matched mentees and 65% of the unmatched mentees earning $100,000 or less, *Χ*^*2*^(6) = 1.61, *p* = .95.

### Quality of life

An independent sample *t*-test was conducted to compare the matched and unmatched mentees on eating disorder quality of life indices. Details of these results can be viewed in Table 2. A comparison of the matched and unmatched mentees revealed that matched mentees reported significantly higher levels of quality of life in the education/vocation, family and close relationships, future outlook, psychological, emotional, values and beliefs, and physical domains than unmatched mentees. It is important to note that mentors’ scores on the EDQLS are consistent with individuals who report low eating disorder symptoms and high levels of functioning [32]. In contrast, both matched and unmatched mentees are reporting quality of life levels consistent with those with high eating disorder symptoms and overall psychiatric symptom severity [32].

**Table 2:** **Group comparisons on eating disorder quality of life scale**

**Quality of life subscales**	**Mentors**	**Matched mentees**	**Unmatched mentees**	***t***	***p***
Cognitive	11.21 (4.28)	7.09 (2.72)	6.45 (3.52)	1.06	.29
Education/vocational	11.42 (4.36)	6.60 (3.38)	5.22 (3.33)	2.12	.04
Family & close relations	12.36 (4.27)	9.26 (3.43)	7.41 (4.11)	2.54	.01
Relationship with others	10.36 (4.05)	7.05 (3.06)	5.86 (3.48)	1.89	.06
Future outlook	12.00 (4.39)	9.43 (3.61)	7.20 (4.36)	2.89	.01
Appearance	10.73 (4.38)	6.31 (3.04)	5.39 (3.35)	1.49	.14
Psychological	10.42 (3.87)	7.38 (2.75)	5.76 (3.50)	2.69	.01
Emotional	9.91 (4.10)	6.52 (2.87)	5.27 (3.33)	2.09	.04
Values & beliefs	10.49 (4.06)	6.64 (2.86)	5.06 (3.18)	2.69	.01
Leisure	11.36 (4.13)	8.62 (3.16)	7.59 (4.15)	1.46	.15
Physical	10.12 (3.79)	7.02 (2.87)	5.69 (3.32)	2.21	.03
Eating	25.52 (10.42)	15.29 (6.51)	13.20 (7.68)	1.52	.13
Total score	145.91 (53.54)	97.24 (34.92)	79.78 (43.05)	2.32	.02

Note: Group comparisons are only between matched and unmatched mentees. Information in the mentor, matched, and unmatched mentee columns is reported in Means (Standard Deviations). *N* = 107, and *df =105* for all comparisons.

### Motivation, energy, and confidence towards recovery

Matched and unmatched mentees were compared on motivation to recover, but no differences were found between the groups, *t* (105) = 0.32, *p* = .75, with both the matched (*M* = 3.98, *SD* = 0.98) and unmatched (*M* = 3.91, *SD* = 1.11) mentees reporting being “somewhat” to “very” motivated to recover. There were no differences between the groups on amount of energy spent towards recovery on a daily basis, *t* (105) = 1.14, *p* = .26, with both the matched (*M* = 3.45, *SD* = 0.98) and unmatched (*M* = 3.22, *SD* = 1.05) mentees reporting spending a considerable amount of energy towards recovery. In addition, there were no differences between the groups on level of confidence that recovery will be achieved, *t* (105) = 0.63, *p* = .53, with both the matched (*M* = 3.16, *SD* = 1.30) and unmatched (*M* = 3.00, *SD* = 1.22) reporting being “fairly” confident that they will recover.

### Treatment compliance

Participants reported the number of missed sessions over the past month. When comparing the matched and unmatched mentees, matched mentees reported missing fewer treatment appointments than unmatched mentees, *t* (105) = 4.83, *p* < .001. Matched mentees reported missing on average 0.98 (*SD* = 1.02) appointments with a range of 0 to 4 sessions and a mode of 1. Unmatched mentees reported missing on average 2.14 (S*D* = 1.40) appointments with a range of 0 to 5 sessions and a mode of 3.

### Time and frequency of communication

Frequency of communication between matched mentees and mentors varied greatly from “2-3 times daily” to “once a month”, with the average being “2-3 times per week” (*M* = 1.74, *SD* = 2.12). Matched mentees were assessed on the length of time they had been in their mentoring relationship and the frequency of communication with their mentors. Pearson product–moment correlations were computed between length of time and frequency of communication, and those variables that emerged significant in the comparisons reported above. As Table 3 displays, the length of time in a mentoring relationship was not significantly associated with any of the quality of life domains, nor motivation, energy or confidence towards recovery. Frequency of communication with your mentor was positively and significantly associated with higher levels of quality of life in the education/vocation, family and close relationships, future outlook, psychological, emotional, values and beliefs, and physical domains.

**Table 3:** **Associations between time, frequency of communication with mentors, and quality of life variables among matched mentees**

	**Length of time**	**Freq of communication**
Motivation	0.02	0.18
Energy	−0.04	0.08
Confidence	0.02	0.23
Education/Vocation	0.20	0.31*
Family & close Rel.	0.04	0.37**
Future outlook	0.03	0.34**
Psychological	0.11	0.40**
Emotional	0.11	0.31*
Values & beliefs	0.13	0.29*
Physical	0.14	0.26*

Note: Table displays pearson correlation coefficients; *p < .05, **p < .01; *N* = 58.

### The mentoring process

Mentors and matched mentees were asked about what topics were frequently discussed by the pairs. Table 4 lists the top topics endorsed by both mentors and matched mentees. In addition to the topics listed in Table 4, mentors also reported frequently discussing treatment compliance, lack of access to professional treatment, lack of willingness to seek treatment, and dealing with weight stabilization. Both matched and unmatched mentees were also asked about their current needs for support services, and Table 5 displays the percentages of the mentees reporting a need for each topic. Approximately one quarter of the sample reported needing support services for learning to name, feel, and deal with emotions. In addition, the other top most pressing needs were dealing with co-occurring issues such as addiction, trauma, depression, etc., and weight stabilization, following a meal plan, and grocery shopping/eating out/food-based socializing.

**Table 4:** **Top list of topics mentors and matched mentees both report discussing the most**

**Topic**	**% Endorsing**
1. Managing or reducing eating disorder thoughts and behaviors	21
2. Staying motivated and committed to doing the hard work of recovery	19
3. Managing relapses	17
4. Choosing recovery	17
5. Setting and working towards recovery goals	16
6. Dealing with triggers from family and environment	16

**Table 5:** **The reported need of both matched and unmatched mentees**

**Topic**	**% Endorsing need**
1. Dealing with triggers from family and environment	17
2. Managing or reducing eating disorder thought and behaviors	14
3. Finding enough quality support services	19
4. Lack of access to professional treatment	14
5. Lack of willingness to seek treatment	11
6. Lack of understanding or support from family and friends	11
7. Support offered by family and friends isn’t what is needed	12
8. Managing relationships with peers, family, friends, colleagues, society	17
9. Asking for and accepting help from others	18
10. Choosing who to ask for help	15
11. Managing relapses	18
12. Body dissatisfaction and poor body image	20
13. Setting and working towards recovery goals	18
14. Dealing with weight stabilization	22
15. Following a meal plan	22
16. Grocery shopping/eating out/food-based socializing	22
17. Learning to name, feel, and deal with emotions	24
18. Finding time for recovery amidst other obligations	20
19. Setting and honoring boundaries	13
20. Choosing recovery	14
21. Finding reasons to recover	16
22. Using my voice and expressing needs, wants, desires, and boundaries	16
23. Staying motivated and committed to doing the hard work of recovery	12
24. Intimacy and relationship challenges, fears, and issues	19
25. Finding and expressing preferences, skills, talents, and identity outside of “Ed”	17
26. Building meaning and quality of life into the recovery process	20
27. Other co-occurring recovery issues with addiction, trauma, abuse, self-harm, depression, anxiety, etc.	23

*N*=107.

### Impact of program on mentors

Mentors were asked an open-ended question about the impact of mentoring on them of which 23 out of 34 mentors answered the question. Overall, 91% of mentors believe the mentoring process positively impacted their own recovery process with three main themes emerging from their responses. They were positively impacted by strengthening the skills they learned while in recovery, solidifying the steps required towards recovery, and reminders to them of how far they’ve come in their own recovery/the unhealthy place they were with the eating disorder.

## Discussion

The overall objective of the present study was to examine if a mentoring relationship improved motivation, confidence, and energy towards recovery for individuals suffering from an eating disorder, and quality of life. Compared to unmatched mentees, mentees matched with a mentor reported significant improvement in 7 of the 12 domains of quality of life including family and close relationships, future outlook, psychological, and emotional domains. In addition, the frequency of communication between mentors and mentees was positively correlated with the same 7 domains of quality of life. There were no differences on motivation, energy or confidence toward recovery between the two groups, with both groups reporting being fairly motivated, confident and spending a considerable amount of energy on a daily basis towards recovery. Interestingly, matched mentees reported more treatment compliance than unmatched mentees, with treatment compliance being assessed as number of missed appointments with treatment providers in the past month. In addition, mentors reported believing that the mentoring relationship further facilitated their own recovery process. The results from the current study corroborate previous research suggesting that there is a significant amount of need for ancillary support services for individuals recovering from an eating disorder [2,3]. Indeed, all of the 27 discussion topics offered to mentees were endorsed. The most pressing remaining unmet recovery support needs identified by mentees were support services for managing co-occurring issues such as addiction, depression, anxiety, trauma, abuse, and self-harm.

The findings from this study suggest that a mentor model could be a beneficial ancillary support service to reinforce treatment goals, with matched mentees reporting higher rates of quality of life and treatment compliance. Quality of life is an important outcome in eating disorder treatment and relapse prevention, and is reported as personally important to patients [32–34]. Matched mentees also reported significant improvement on family and close relationships domain, indicating higher levels of perceived social support. Increased social support is important towards treatment outcome because low levels of perceived social support during negative life events can exacerbate eating disorder symptoms [35]. The benefits of the mentor model were further corroborated with more frequent communication between mentors and mentees being positively associated with higher scores in the quality of life domains. In addition, 91% of the mentors that completed the open-ended questions reported that the mentoring relationship positively impacted their own recovery process, supporting the helper therapy principle which states that there are intrinsic rewards and benefits to helpers when they assist others, including maintaining treatment outcomes [27].

There were several programmatic components to implementing a mentor program that warrant discussion. First, we suggest assigning an experienced mentor to a new mentor to answer any questions and facilitate the new mentor’s process of learning to become a confident and effective mentor. The mentoring relationship can be a different type of relationship and navigating those boundaries can be facilitated with an experienced mentor as a coach particularly for those mentors who are treatment professionals and can have difficulty defining the boundary between a supportive and a therapeutic relationship. The mentor program was structured as a strictly non-medical, non-clinical source of supplemental peer-based support that can complement but never substitute nor replace the vital work of the treatment professional. Thus, there is a strong emphasis to the mentor program for enhancing recovery and treatment results, but in such a way as to conscientiously refrain from interfering with treatment. Examples of this were evident in the responses from mentors on open-ended questions regarding what they focused on and discussed during their conversations with mentees. For example, under the topic “setting and working toward recovery goals,” a frequent theme among mentors with mentees suffering from anorexia nervosa was honesty with treatment providers, with mentors learning that at times their mentees withheld information from their treatment providers and worked with their mentees on being more honest to maximize treatment gains. In sum, a clear definition of a mentor relationship and the boundaries of that relationship are hypothesized to be an important component of the program. However this has not been tested and future research needs to investigate the key components of a mentor model.

Past research has reported that professionals have some reservations about recovered individuals being involved in the recovery process of recovering persons [25]. The findings from this study suggest that a mentor model that includes recovered individuals as mentors is beneficial to individuals in the recovery process of an eating disorder. Aside from the current study, there has been some research, albeit limited, that has investigated the role of recovered individuals in facilitating recovery in others. A couple of studies have found that approximately one-third of treatment providers in the eating disorders field have recovered from an eating disorder [36,37]. Costin and colleagues [25,26,38] have outlined the benefits of having recovered individuals involved in the treatment of others. Specifically, patients and carers consider the experience of a recovered person to be a positive asset in the quality of the relationship and advice given [25]. However, concerns of having recovered individuals involved in the recovery process of others mainly stem around risk of relapse [25,38], and Costin and colleagues highlight that the longer a person is recovered and views themselves as recovered, might reduce risk of relapse [26].

There are several limitations of this study that are worth noting. First, this is a cross-sectional study and a preliminary investigation which limits the findings to correlational associations. It is possible that the higher quality of life functioning and treatment compliance among the matched mentees was due to some confounding variable not measured by this study. It is also unknown if matched and unmatched mentees differed on personality characteristics or some other variables that could have potentially made a mentee more or less likely to find a mentor. Another limitation is the sample, which consisted of matched and unmatched mentees, limiting the generalizability of the findings. Mentees were individuals who self-identified as needing additional support for recovery from an eating disorder and were motivated enough to join and actively search for a mentor. Thus, the entire sample consisted of individuals that had demonstrated a significant level of motivation and action towards recovery, and the findings from this study may not apply to individuals in the contemplation or pre-contemplation phases of treatment. In addition, a significant number of members at MentorCONNECT did not participate in this study, thus raising the possibility that our sample is biased or unrepresentative.

This investigation into a mentor program for eating disorders is preliminary, and needs replication. The beneficial findings of this study will hopefully ignite future research. Indeed the ancillary support services literature for eating disorders is in need of empirical research that examines the benefits of these services and for whom. Future research could address who is most likely to benefit from matching with a mentor, what models of mentorship will yield better results, and what are the essential components of a successful mentor-mentee match.

## Conclusion

The findings from this study indicate that a mentor program significantly improves the quality of life and treatment compliance for individuals working towards recovery. The potential benefits of a mentor program in alleviating some of the immediate and pressing needs for additional recovery-based support services and the positive findings in this study suggest that mentor programs warrant further investigation.
